# Burden, trends, and predictions of low physical activity-related diseases in China: analysis from the Global Burden of Disease Study, 1990–2021, with projections to 2035

**DOI:** 10.3389/fpubh.2025.1461554

**Published:** 2025-02-28

**Authors:** Chuan Zhang, Quanzheng Chen, Fuqiang Yin, Lanyan Qin, Shuna Zhang

**Affiliations:** ^1^Department of Physical Education and Health, Guangxi Normal University, Guilin, China; ^2^Department of Graduate, Wuhan Sport University, Wuhan, China; ^3^Department of Graduate, Xiangnan University, Chenzhou, China

**Keywords:** low physical activity, disease burden, Global Burden of Disease (GBD) database, Bayesian Age-Period-Cohort model (BAPC), estimated annual percentage change, trends analysis

## Abstract

**Objective:**

To evaluate the burden of disease related to low physical activity in China, examine its trends over time, and predict future trends up to 2035 to inform preventive actions.

**Methods:**

Using Global Burden of Disease Study 2021 (GBD2021) data, we analyzed the burden of disease associated with low physical activity, employing Deaths and Disability-Adjusted Life Years (DALYs) as indicators. Trends from 1990 to 2021 were examined using the Estimated Annual Percentage Change (EAPC) method, and future projections were made using the BAPC model.

**Results:**

The number of deaths attributable to low physical activity rose from 48,882 in 1990 to 148,152 in 2021, with the age-standardized death rate decreasing slightly from 8.39 to 8.18 per 100,000 (EAPC = 0.07). DALYs increased from 1,246,888 to 3,254,644, while the age-standardized DALYs rate fell from 168.93 to 162.52 per 100,000 (EAPC = −0.08). The DALY rate for diabetes increased from 30.12 to 36.54 per 100,000 (EAPC = 0.42); ischemic heart disease death rate rose from 2.86 to 3.52 per 100,000 (EAPC = 1.12), and the DALY rate increased from 40.72 to 46.05 per 100,000 (EAPC = 0.8). The highest death and DALY rates were observed in individuals over 95 years old. Females had higher death and DALY rates than males, with a death rate of 8.29 per 100,000 and a DALY rate of 197.98 in 2021. The BAPC model predicts that by 2035, deaths will reach 161,280 and DALYs will reach 3,523,135.

**Conclusion:**

From 1990 to 2021, the age-standardized death and DALY rates associated with low physical activity in China declined, yet the absolute number of deaths and DALYs increased due to population growth and aging. Special attention is needed for diabetes and ischemic heart disease, as their burdens have significantly risen. Although females are more affected by low physical activity than males, the increase in the burden among males has been more pronounced in recent years. The impact of low physical activity intensifies with age, particularly among the older adult. Thus, targeted strategies are essential to mitigate the burden of low physical activity in China.

## 1 Introduction

Low physical activity has emerged as one of the most formidable challenges in global public health today. According to World Health Organization statistics, approximately 3.5 million deaths annually are attributed to low physical activity, resulting in a reduction of life expectancy by 1.5 to 3 years, establishing it as the fourth leading risk factor for mortality worldwide (1, 2). In academic research, the conceptual definition of low physical activity has undergone an evolution from simplistic to intricate interpretations. The World Health Organization (WHO) employs a foundational definition, characterizing it as falling short of either 150 min of moderate-intensity aerobic activity or 75 min of vigorous activity per week (3, 4). The GBD project, however, has developed a more comprehensive assessment framework, integrating dimensions such as activity type, frequency, and intensity, while combining self-reported data with objective monitoring metrics, utilizing indicators like Metabolic Equivalent of Task (MET) to evaluate the impact of low physical activity more thoroughly (5). In this study, we adopted the GBD project’s definition and assessment framework for low physical activity, which provides more comprehensive and objective measurements compared to the WHO’s basic threshold. The GBD framework considers multiple dimensions: (1) activity intensity measured by METs, where moderate-intensity activities are defined as 3–6 METs and vigorous activities as >6 METs; (2) activity frequency and duration, requiring a minimum of 10 consecutive minutes per session; and (3) activity type, including work-related physical activity, transportation, household tasks, and leisure-time physical activities. This integrated approach allows for a more accurate assessment of the actual physical activity levels and their health impacts (5, 6).

Low physical activity has imposed a substantial disease burden on major global economies. In developed nations, approximately 50% of American adults fail to meet recommended activity levels, resulting in annual healthcare expenditures exceeding $117 billion; similarly, about 40% of British adults are insufficiently active, incurring direct medical costs of roughly £1 billion per year. In China, the health burden associated with low physical activity is particularly severe. Data indicates that around 500,000 deaths annually are due to low physical activity, ranking it as the fifth leading risk factor for death, following hypertension, smoking, high-salt diet, and air pollution (7, 8). With China’s aging population and changing lifestyles, the disease burden from low physical activity is projected to increase, placing additional strain on economic, social, and healthcare systems (9–11). Existing research primarily focuses on the physical activity patterns and health impacts among various demographics (such as adults, the older adult, children, and adolescents), as well as the differences between urban and rural areas and environmental factors influencing physical activity. However, there has been no systematic analysis of the burden caused by low physical activity specifically in China (6, 12–15). This study utilizes data from the GBD 2021 dataset to provide a comprehensive analysis of the burden of low physical activity in China from 1990 to 2021, examining trends by age, gender, and disease category, and projecting the burden for the next 14 years. By leveraging GBD data, this study aims to provide policymakers, researchers, and the public with detailed insights into the state of low physical activity in China, facilitating the development of more effective targeted policies and interventions.

## 2 Data and methods

### 2.1 Study data

The data for this study is sourced from the GBD 2021. The GBD project, in collaboration with the WHO, the World Bank, the Bill & Melinda Gates Foundation, and various academic institutions, research organizations, and national governments, systematically evaluates global and regional disease burdens and their trends. Initiated in 1990, the GBD study aims to provide scientific evidence for health policy formulation and resource allocation. The GBD database is actively maintained and updated using research data, published epidemiological studies, and government publications from over 90,000 sources. GBD 2021 employs a globally standardized methodology to estimate key indicators such as incidence, prevalence, Death, Years of Life Lost (YLL), Years Lived with Disability (YLD), and total DALY for 204 countries and territories (16, 17). These estimates, which include 95% Uncertainty Intervals (UI), ensure the reliability and accuracy of the data and provide essential insights for mitigating disease burdens globally and locally.

The Global Burden of Disease (GBD) 2021 database is the sole data source for this study. While the GBD database contains data from 204 countries and regions, our primary analysis focuses exclusively on data from mainland China. To contextualize China’s disease burden, we include global comparisons in Section 3.3 (‘Differences between China and the global average’), where we analyze how China’s disease burden due to low physical activity compares with global averages across different age groups and time periods. Additionally, while the data covers the entire Chinese population and provides nationwide disease burden estimates, the GBD database does not classify China into urban and rural areas separately. Therefore, the data analysis in this study is based on the entire population of China without further differentiation between urban and rural regions.

In the GBD 2021 dataset, disease burden is classified into different hierarchical levels. Since the initiation of the GBD project in 1990, all versions, including GBD 2017 and GBD 2019, have utilized the same hierarchical classification system. Level 1 categories include communicable diseases, non-communicable diseases, and injuries. Level 2 further divides these into broader disease groups, such as cardiovascular diseases and respiratory diseases. Level 3, in turn, includes more specific diseases and causes, such as ischemic heart disease and stroke. This study focuses on the analysis of Level 3 diseases (see Table 1) to provide a more detailed understanding of the burden and trend changes associated with specific diseases (18). Moreover, the study found that both mortality and the number of DALYs attributable to low physical activity were zero for individuals under 25 years of age. Therefore, data for those under 25 years were not included in this study. All other age groups were included, and burden trends were derived based on data from these age categories.

**Table 1:** Statistical models used in the study and their specifications.

Model	Purpose	Parameters	Data input
EAPC (estimated annual percentage change)	Analyze temporal trends (1990–2021)	Formula: y = α + βx + *ε* EAPC = (exp(*β*) − 1) × 100% β (regression coefficient) Calendar year (x) Log of ASR (y)	Age-standardized rates from adults aged 25 and older Annual time points of low physical activity from 1990 to 2021
BAPC (Bayesian Age-Period-Cohort)	Predict sex-specific Death and DALYs (2021–2035)	Formula: log(λ_ijt) = μ + *α*_i + β_j + γ_k + ε_ijt Age effect Period effect Cohort effect INLA parameters for Bayesian inference	Historical counts of physical activity cases from 1990 to 2021 Age-specific rates for adults aged 25 and older Gender-specific data for males and females separately

EAPC = Estimated Annual Percentage Change; ASR = Age-Standardized Rate; BAPC = Bayesian Age-Period-Cohort; INLA = Integrated Nested Laplace Approximations; Data Source: All data were obtained from the Global Burden of Disease Study 2021 (GBD 2021) through the GBD Results Tool (http://ghdx.healthdata.org/gbd-results-tool).

### 2.2 The model and model input

Two statistical models were employed in this study. The first model, the Estimated Annual Percentage Change (EAPC), was used to describe long-term trends of various diseases caused by low physical activity from 1990 to 2021. EAPC provides a quantitative measure of the average rate of change per year over a specified time interval (5, 19). Trends are considered to be increasing if both the EAPC value and the lower limit of the 95% UI are greater than 0, decreasing if both the EAPC value and the upper limit of the 95% UI are less than 0, and constant if the 95% UI includes 0 (17).

The second model, to predict sex-specific mortality and DALY numbers from 2021 to 2035, we employed the Bayesian Age-Period-Cohort (BAPC) model. BAPC model is capable of capturing the influence of age, period, and birth cohort on disease burden, making it particularly suitable for analyzing trends over time. Through combining Bayesian inference with age, period and cohort interaction effects, this model enables more accurate predictions of future burden changes, as shown in Table 1.

### 2.3 Data analysis

The data analysis in this study was conducted through multiple steps. First, we used Office Excel 2019 to organize and calculate age-standardized death rates (ASDR) and age-standardized DALY rates. To avoid bias from different population structures, we employed the standard population age structure to calculate attributable death and DALY rates, with results presented as age-standardized rates per 100,000 population.

Subsequently, we analyzed the distribution of disease burden across different population groups. In the age-specific analysis, we focused on age groups 25 years and older, as both mortality and DALYs attributable to low physical activity were zero for individuals under 25 years. We conducted sex-specific analyses to examine differential impacts between males and females. Additionally, we performed temporal trend analyses, tracking changes in disease burden from 1990 to 2021, with projections to 2035.

For comprehensive visualization and statistical analysis, we utilized Python and R (version 4.3.2), along with packages such as ggplot2 and maps. These tools enabled us to generate various visualizations and conduct detailed statistical analyses of burden trends. All statistical analyses included 95% Uncertainty Intervals (UI) to account for potential variability in estimates. The detailed code for all analyses and visualizations is available on GitHub.^1^

## 3 Results

### 3.1 Global burden of disease and trends attributable to low physical activity

Table 2 presents the comprehensive data on Deaths, DALYs, and their respective age-standardized rates for low physical activity in China from 1990 to 2021. The total number of deaths due to low physical activity increased from 48,882.07 (95% UI: 20,381.00 to 81,461.04) in 1990 to 148,151.71 (95% UI: 57,143.15 to 264,484.08) in 2021. The age-standardized death rate showed a slight decrease from 8.39 (95% UI: 3.18 to 14.94) to 8.18 (95% UI: 2.94 to 14.95) per 100,000, with an EAPC of 0.07 (95% UI: −0.14 to 0.28). The number of DALYs increased from 1,246,888.39 (95% UI: 526,730.42 to 1,981,540.08) to 3,254,643.75 (95% UI: 1,383,116.10 to 5,324,092.47), while the age-standardized DALY rate declined from 168.93 (95% UI: 70.81 to 276.67) to 162.52 (95% UI: 66.38 to 269.91) per 100,000, with an EAPC of −0.08 (95% UI: −0.21 to 0.04).

**Table 2:** Death rate, number of deaths, DALY rate, number of DALYs, and temporal trends (EAPC) from 1990 to 2021 attributable to low physical activity in 1990 and 2021.

Causes	DALYs (95% UI)					Deaths (95% UI)				
	1990		2021		Eapc	1990		2021		Eapc
	Number	Rate	Number	Rate		Number	Rate	Number	Rate	
All causes	1246888.39 (526730.42 to 1981540.08)	168.93 (70.81 to 276.67)	3254643.75 (1383116.10 to 5324092.47)	162.52 (66.38 to 269.91)	−0.08 (−0.21 to 0.04)	48882.07 (20381.00 to 81461.04)	8.38 (3.18 to 14.94)	148151.71 (57143.15 to 264484.08)	8.18 (2.94 to 14.95)	0.07 (−0.14 to 0.28)
Breast cancer	23212.51 (4512.08 to 42571.46)	2.56 (0.50 to 4.76)	54895.50 (10086.19 to 103414.52)	2.61 (0.47 to 4.91)	−0.23 (−0.34 to −0.12)	748.38 (144.91 to 1399.42)	0.10 (0.02 to 0.18)	1925.20 (371.12 to 3598.35)	0.09 (0.02 to 0.17)	−0.49 (−0.62 to −0.35)
Chronic kidney disease	78751.33 (31192.37 to 141575.01)	11.04 (4.45 to 19.52)	189095.11 (72233.55 to 332756.05)	9.43 (3.59 to 16.48)	−0.56 (−0.71 to −0.41)	3038.92 (1216.42 to 5419.25)	0.55 (0.23 to 0.97)	8700.75 (3312.81 to 15144.77)	0.48 (0.19 to 0.83)	−0.58 (−0.72 to −0.45)
Colon and rectum cancer	128352.60 (74846.96 to 188831.95)	17.12 (10.06 to 24.93)	320464.35 (192274.95 to 474069.92)	15.63 (9.47 to 22.88)	−0.4 (−0.52 to −0.28)	5734.54 (3368.81 to 8371.03)	0.93 (0.55 to 1.36)	16698.47 (10065.03 to 24625.76)	0.87 (0.53 to 1.29)	−0.33 (−0.41 to −0.25)
Diabetes mellitus	234966.16 (99510.02 to 371960.19)	30.12 (12.64 to 46.95)	757445.48 (327374.89 to 1219478.84)	36.54 (15.85 to 58.04)	0.42 (0.29 to 0.54)	5532.07 (2274.51 to 8709.07)	0.89 (0.37 to 1.40)	17106.39 (7495.19 to 28029.00)	0.90 (0.40 to 1.47)	0.02 (−0.24 to 0.28)
Ischemic heart disease	237710.36 (103505.75 to 396304.46)	40.72 (16.75 to 71.08)	827789.03 (365854.70 to 1470328.11)	46.05 (19.74 to 83.48)	0.8 (0.52 to 1.07)	12808.26 (5227.16 to 22545.48)	2.86 (1.12 to 5.37)	57720.55 (23735.00 to 106093.78)	3.52 (1.43 to 6.58)	1.12 (0.78 to 1.46)
Lower extremity peripheral arterial disease	1841.09 (436.14 to 4082.36)	0.27 (0.07 to 0.60)	5461.43 (1286.88 to 11834.63)	0.27 (0.06 to 0.58)	−0.45 (−0.62 to −0.29)	15.68 (4.27 to 31.96)	0.00 (0.00 to 0.01)	71.37 (20.05 to 145.63)	0.00 (0.00 to 0.01)	0.62 (0.42 to 0.83)
Stroke	510439.38 (186946.39 to 891930.65)	63.24 (11.05 to 124.92)	1086837.93 (230331.06 to 2144873.82)	51.38 (6.16 to 108.84)	−0.7 (−0.83 to −0.57)	19802.84 (3684.80 to 38636.46)	2.88 (−0.90 to 6.95)	45502.96 (−9993.95 to 112130.22)	2.29 (−1.04 to 6.20)	−0.75 (−0.95 to −0.55)
Tuberculosis	31614.96 (9580.25 to 60368.81)	3.86 (1.22 to 7.43)	12654.93 (3926.63 to 24167.23)	0.60 (0.19 to 1.16)	−6.14 (−6.34 to −5.93)	1201.38 (382.79 to 2309.89)	0.17 (0.05 to 0.33)	426.01 (133.07 to 817.77)	0.02 (0.01 to 0.04)	−7 (−7.27 to −6.72)

DALYs = Disability-Adjusted Life Years; UI = Uncertainty Intervals; EAPC = Estimated Annual Percentage Change; Rate = per 100,000 population. Values in parentheses represent 95% UI. The number of deaths and DALYs are presented as absolute values, while rates are age-standardized per 100,000 population.

**Figure 1:**
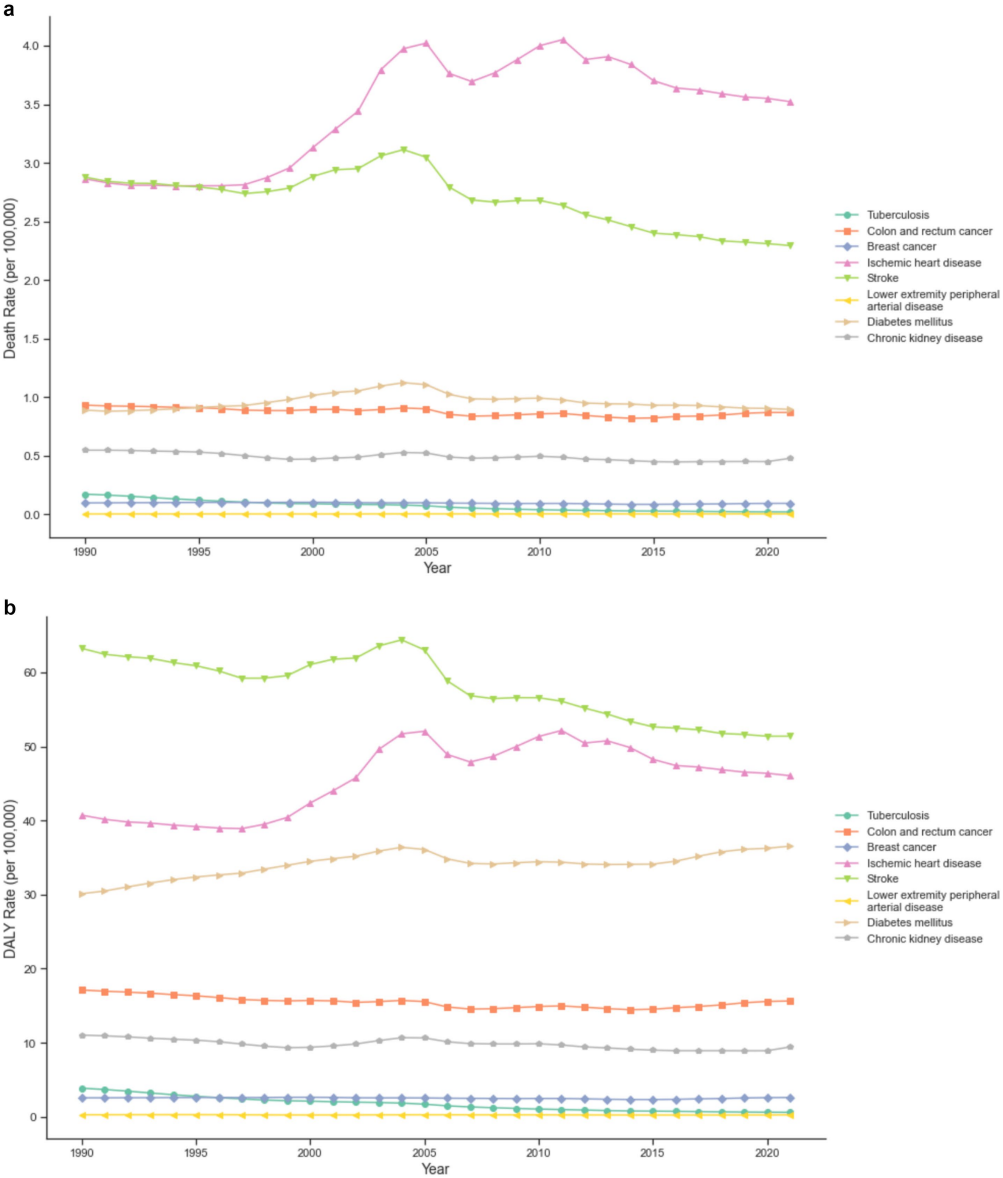
**(a)** Trends in death rates for Level 3 major diseases attributable to low physical activity in China from 1990 to 2021. **(b)** Trends in DALY rates for Level 3 major diseases attributable to low physical activity in China from 1990 to 2021.

As shown in Figures 1a,b, among various diseases, three conditions showed notable trends. The stroke death rate decreased from 2.88 (95% UI: −0.90 to 6.95) to 2.29 (95% UI: −1.04 to 6.20) per 100,000, with an EAPC of −0.75 (95% UI: −0.95 to −0.55). For diabetes mellitus, while the death rate remained stable with an EAPC of 0.02 (95% UI: −0.24 to 0.28), the DALY rate increased significantly from 30.12 (95% UI: 12.64 to 46.95) to 36.54 (95% UI: 15.85 to 58.04) per 100,000. Ischemic heart disease showed the most concerning trend, with the death rate increasing from 2.86 (95% UI: 1.12 to 5.37) to 3.52 (95% UI: 1.43 to 6.58) per 100,000, and DALY rate rising from 40.72 (95% UI: 16.75 to 71.08) to 46.05 (95% UI: 19.74 to 83.48) per 100,000.

### 3.2 Burden and trends of diseases caused by low physical activity across different genders and age groups

**Figure 2:**
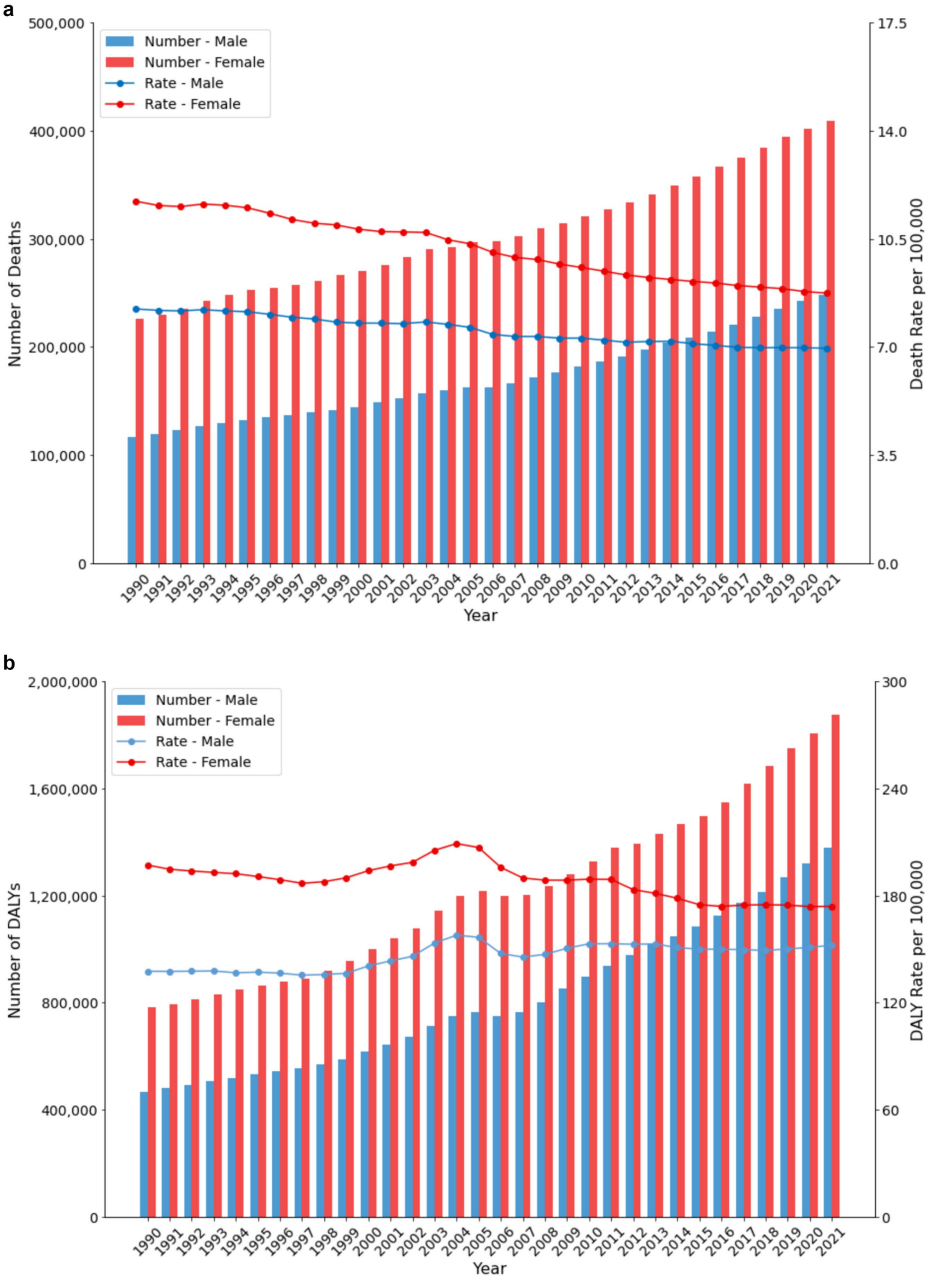
**(a)** Trends in death number and death rate by sex in China (1990–2021). **(b)** Trends in DALY number and DALY rate by sex in China (1990–2021).

Gender analysis revealed distinct patterns in the burden of low physical activity (Figures 2a,b). For females, the death rate decreased from 9.31 (95% UI: 3.65 to 17.13) to 8.29 (95% UI: 2.90 to 16.00) per 100,000, while the number of deaths increased from 31,043.34 (95% UI: 13,277.73 to 53,452.42) to 85,935.75 (95% UI: 31,627.28 to 163,713.00). The female DALY rate decreased from 197.08 (95% UI: 82.59 to 330.26) to 173.98 (95% UI: 72.22 to 300.71) per 100,000. For males, both rates and absolute numbers increased, with the death rate rising from 7.11 (95% UI: 2.04 to 13.69) to 8.22 (95% UI: 2.65 to 16.55) per 100,000, and the DALY rate increasing from 137.57 (95% UI: 50.85 to 246.81) to 152.11 (95% UI: 61.70 to 275.42) per 100,000.

**Figure 3:**
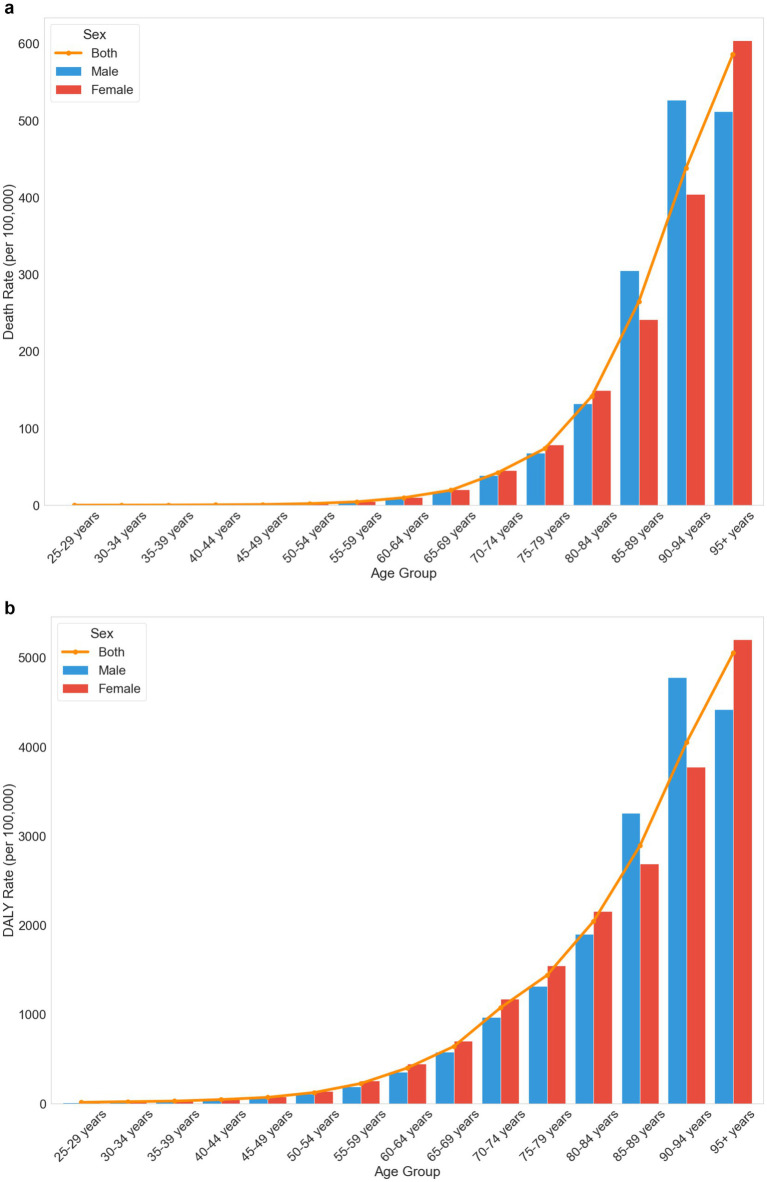
**(a)** Age-and sex-specific trends in death rates attributable to low physical activity in China in 2021. **(b)** Age-and sex-specific trends in DALY rates attributable to low physical activity in China, 2021.

Age-specific analysis (Figures 3a,b) showed that burden intensifies with age, becoming particularly pronounced after age 60. The highest burden was observed in the 95+ age group, with a death rate of 586.37 per 100,000 (95% UI: 176.83 to 1237.61) and a DALY rate of 5052.57 per 100,000 (95% UI: 1525.58 to 10410.6). Notable gender differences emerged in the 85–94 age range, where males showed higher rates than females, with male death rates reaching 526.89 per 100,000 (95% UI: 77.21–1241.55) compared to female rates of 404.08 per 100,000 (95% UI: 96.33–916.70).

### 3.3 Differences between China and the global average

**Figure 4:**
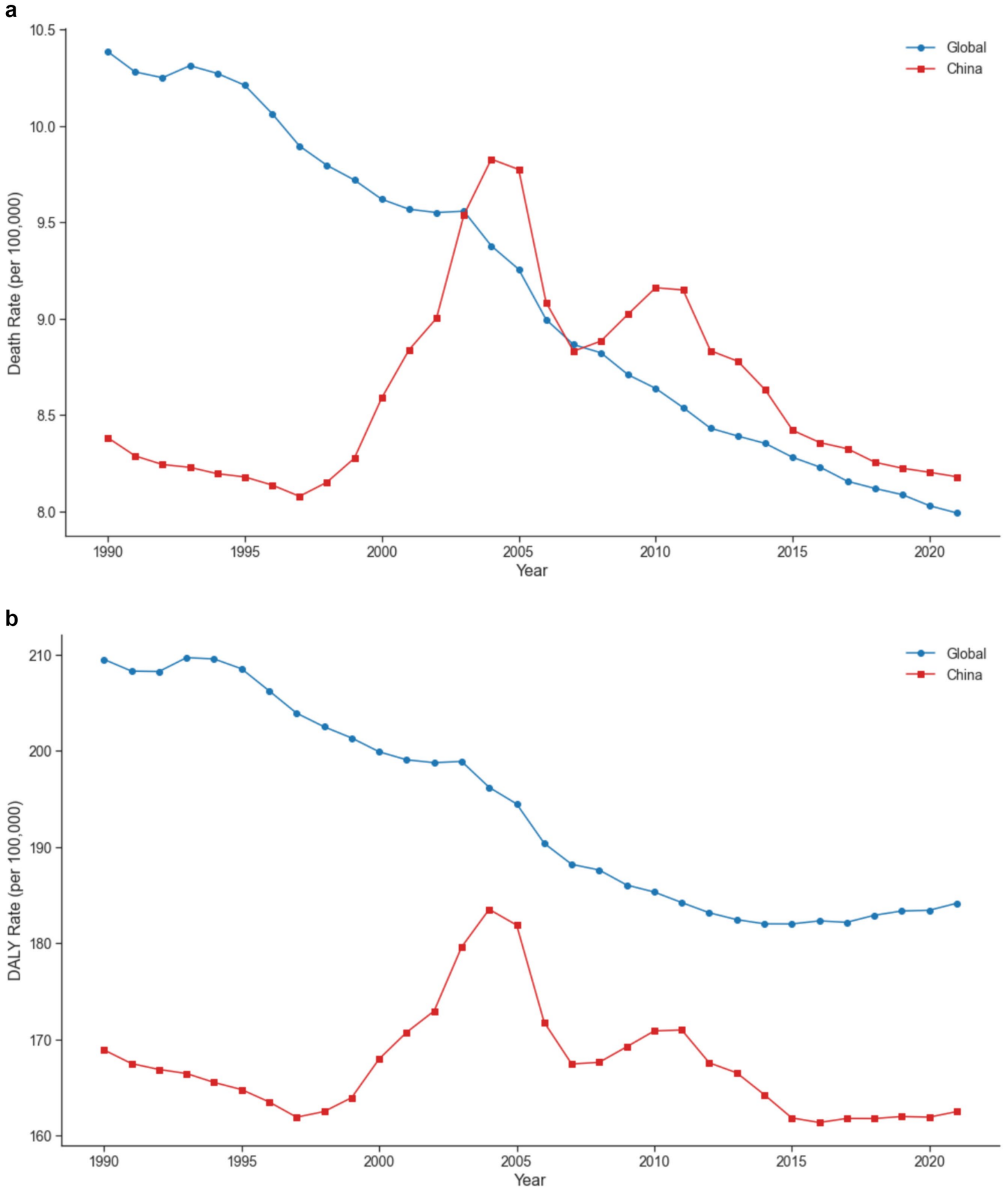
**(a)** Comparative trends in death rates attributable to low physical activity between China and the global average from 1990 to 2021. **(b)** Comparative trends in DALY rates attributable to low physical activity between China and the global average from 1990 to 2021.

Figures 4a,b illustrate the trends in Death and DALY rates due to low physical activity in China and globally from 1990 to 2021. Globally, both rates showed steady declines, with the Death rate decreasing from 10.39 to 7.99 per 100,000 people and the DALY rate from 209.51 to 184.17 per 100,000 people. In contrast, China’s trends showed more fluctuation, particularly during 1997–2004. The Death rate in China declined from 8.38 to 8.18 per 100,000 people, while the DALY rate decreased from 168.93 to 162.52 per 100,000 people over the study period.

**Figure 5:**
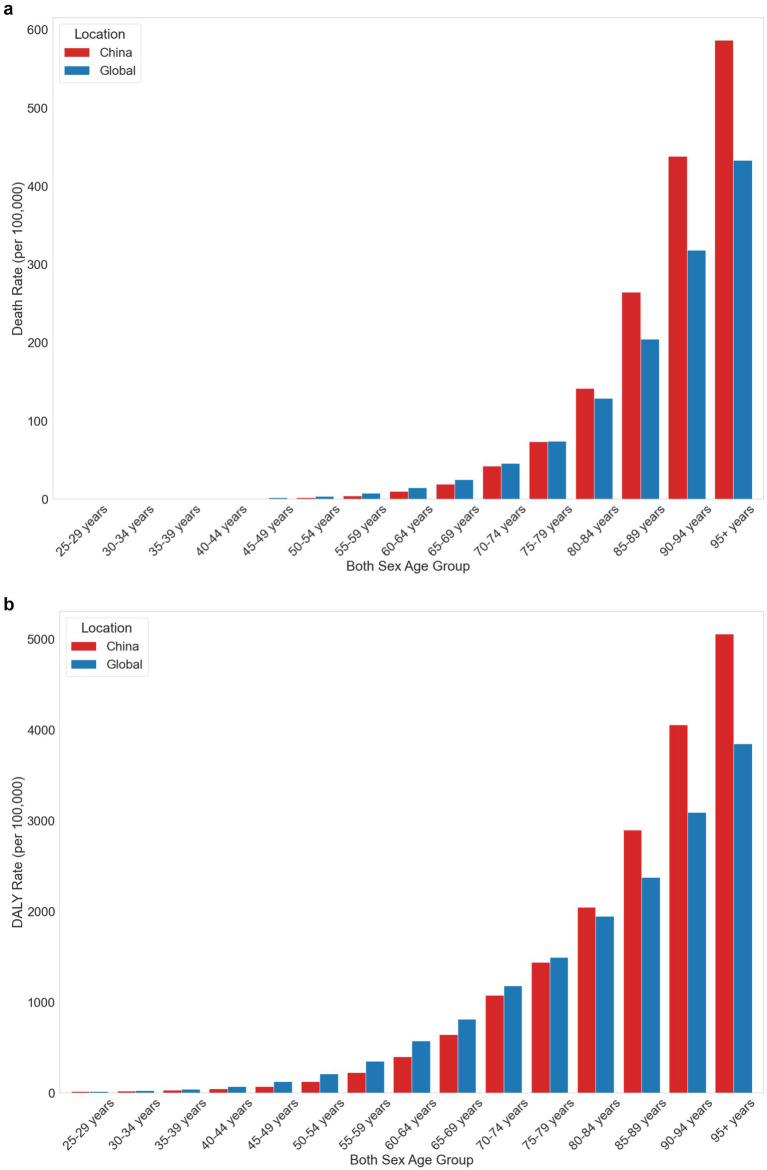
**(a)** Comparison of death rates attributable to low physical activity by age group between China and the global average in 2021. **(b)** Comparison of DALY rates attributable to low physical activity by age group between China and the global average in 2021.

Age-specific comparisons (Figures 5a,b) revealed that differences between China and global rates became more pronounced in older age groups. In the 80–84 age group, China’s Death rate was 141.79 per 100,000 people (95% UI: 36.08 to 297.14) compared to the global rate of 128.66 (95% UI: 45.04 to 227.36). The gap widened in the 95+ age group, with China’s Death rate reaching 586.37 per 100,000 people (95% UI: 176.83 to 1237.61) versus the global rate of 432.85 (95% UI: 147.20 to 777.05). DALY rates showed similar patterns, with China’s rate in the 95+ age group at 5052.57 per 100,000 people (95% UI: 1525.58 to 10410.6) significantly higher than the global rate of 3846.81 (95% UI: 1352.03 to 6936.19).

### 3.4 Projections of DALYs and deaths related to low physical activity in China through 2035

**Figure 6:**
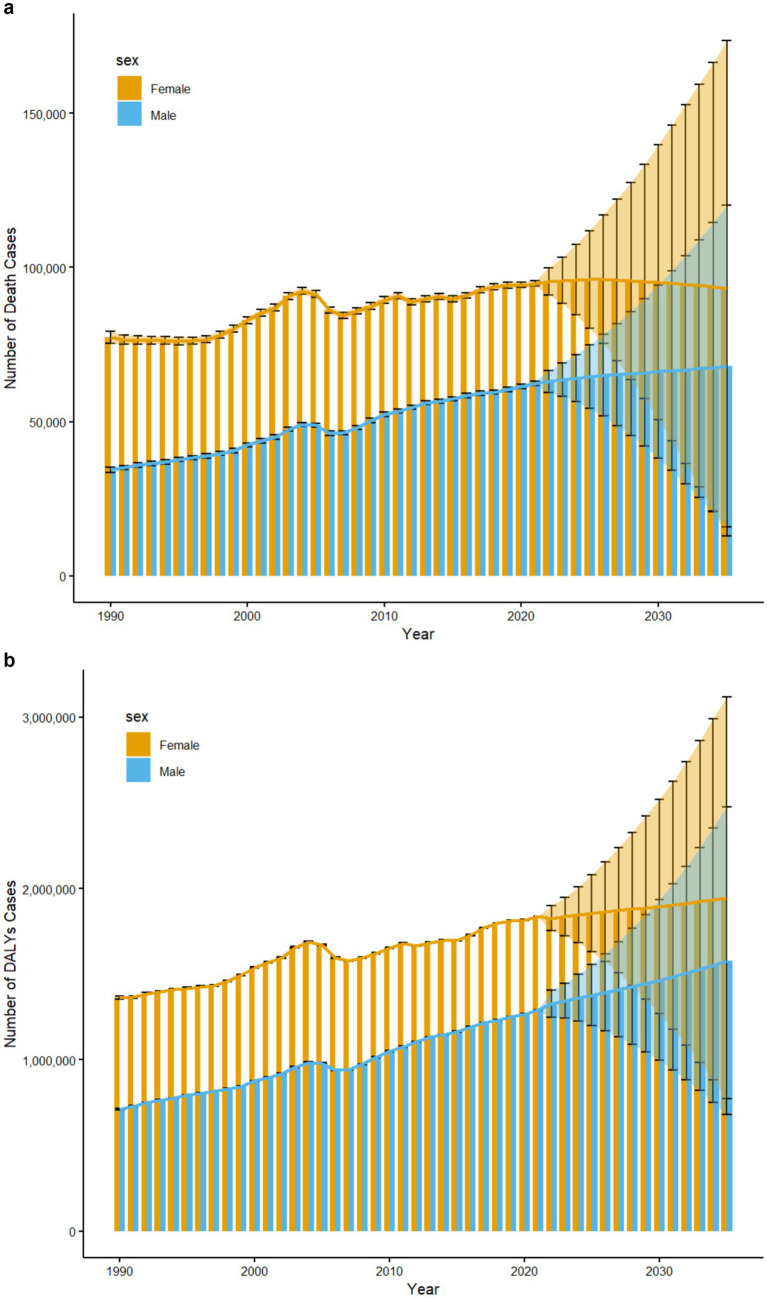
**(a)** Trends in the number of death cases attributable to low physical activity in China from 1990 to 2035, with predictions for 2021–2035 by sex. **(b)** Trends in the number of DALY cases attributable to low physical activity in China from 1990 to 2035, with predictions for 2021–2035 by sex.

Figure 6 forecasts the global trend in the number of deaths and DALYs related to low physical activity by gender through 2035. The overall trend indicates a continuous rise in both deaths and DALYs over the next few decades. By 2035, the total number of deaths is projected to reach 161,279.90, an increase of 4,063.58 from 2021, while the total number of DALYs is expected to reach 3,523,135, an increase of 397,922.90 from 2021.For males, the number of deaths is projected to reach 68,013.66, an increase of 5,606.75 from 2021. In contrast, the number of female deaths is expected to decrease by 1,543.17, reaching 93,266.23. Similarly, the number of male DALYs is anticipated to rise to 1,578,234, an increase of 288,363, while the number of female DALYs will grow to 1,944,901, an increase of 109,559.9.

## 4 Discussion

This study is the first to systematically analyze the impact of low physical activity on the disease burden in China from 1990 to 2021 and to predict future trends. Overall, while the DALY rate and Death rate related to low physical activity have decreased, the number of deaths and DALYs has significantly increased. This increase is primarily due to the rapid population growth in mid-20th century China, which was driven by a stable post-civil war social environment, improved medical conditions, higher agricultural production levels, and supportive policies. As a result, the proportion of older adult people grew substantially by the early 21st century (20). Additionally, advancements in medical and health conditions have significantly increased the average life expectancy in China, rising from 69 years in 1990 to 77 years in 2020, according to WHO data (21). The extended life expectancy, combined with the large population base from the mid-20th century, has exacerbated the aging population issue, leading to a substantial disease burden. This underscores the need for China to enhance its chronic disease management system.

Analyzing the overall time trends, the burden of low physical activity in China shows greater volatility compared to the continuous global decline, with significant increases in Death and DALY rates during the periods of 1997–2004 and 2007–2010. The rise between 1997 and 2004 can be attributed to the substantial economic growth following China’s accession to the WTO. World Bank data indicate that China’s annual GDP growth rate exceeded 8% from 1997 to 2004, leading to a dramatic shift in industrial structure from agriculture and traditional manufacturing to modern services and technology industries. This shift altered work and lifestyle patterns, reducing physical labor (22). Concurrently, the advent of television and the internet redirected entertainment indoors, diminishing the inclination for physical activities (23). The increase from 2007 to 2010 is associated with the rapid economic growth and urbanization driven by economic globalization. During this period, China’s annual GDP growth rate averaged 9.7%, peaking at 14.2% in 2007. The urbanization rate rose from 44.9% in 2007 to 49.9% in 2010, concentrating populations in cities, accelerating the pace of life, and increasing stress levels, which further reduced physical activity. Additionally, private vehicle ownership surged, with a 70% increase in motor vehicles from 7.6 million in 2007 to over 130 million in 2010, significantly reducing opportunities for walking and cycling, thus lowering physical activity levels further (24, 25). To mitigate the adverse effects of low physical activity on health, China must enhance health education and promotion, expand public fitness facilities, advocate for healthier work habits, and encourage green travel. These measures are crucial for effectively reducing the health burden associated with low physical activity and improving overall public health.

When examining specific diseases, China particularly needs to focus on Diabetes mellitus and ischemic heart disease. The burden of these two diseases has significantly increased, contrary to the trends of other diseases. Specifically, the number of DALYs for Diabetes mellitus increased by 522,479 people, which is 3.22 times the number in 1990. The number of deaths from ischemic heart disease increased by approximately 4.51 times. The primary cause of this trend is the rapid urbanization, which has altered lifestyles. For example, the widespread adoption of fast food culture has led to dietary habits high in fat, sugar, and salt. Additionally, advances in modern technology have shifted the labor structure toward more sedentary jobs, significantly increasing the risk of ischemic heart disease and diabetes, thus exacerbating their burden (20, 26). Furthermore, rapid industrialization and environmental pollution have intensified the negative impacts of cardiovascular diseases. Although the Chinese government has implemented policies since 2013 to reduce the annual average PM2.5 concentration by 30% by 2019, the levels remain high. Zhang et al. reported that the annual average PM2.5 concentration in China in 2019 was 48 μg/m^3^, far exceeding the WHO recommended safety standard of 10 μg/m^3^ (27). According to Rajagopalan et al. (28) each 10 μg/m^3^ increase in PM2.5 concentration raises the daily Death rate from cardiovascular diseases by 0.4%. Therefore, China must enhance the prevention and management of low physical activity, particularly through interventions targeting obesity and metabolic disorders, while continuing to advance policies and measures to improve air quality. These actions are crucial for reducing the disease burden associated with low physical activity.

From the perspective of gender differences, low physical activity has a greater negative impact on women than men. Women have higher Death rates, death counts, DALY rates, and DALY counts related to low physical activity. This is primarily because estrogen protects the cardiovascular system, and postmenopausal women are at higher risk for chronic diseases such as hypertension and cardiovascular disease after losing this protection (29). Additionally, postmenopausal women experience a decline in basal metabolic rate, leading to weight gain and abdominal obesity. Flegal et al. found that the obesity rate for women over 65 is about 5% higher than for men, and obesity increases the risk of insulin resistance and type 2 diabetes, contributing to a greater disease burden (30). Additionally, postmenopausal women experience a decline in basal metabolic rate, leading to weight gain and abdominal obesity. Flegal et al. found that the obesity rate for women over 65 is about 5% higher than for men, and obesity increases the risk of insulin resistance and type 2 diabetes, contributing to a greater disease burden. Moreover, the decline in estrogen also affects bone density, muscle mass, and strength, making women more prone to lower back pain and disc herniation, which further hinders physical activity (31). To address these challenges, China needs to develop and promote social policies and public health measures to increase women’s participation in physical activity and improve their health. These measures may include providing more sports facilities, enhancing health education and promotion, raising awareness of women’s health issues, and promoting gender equality. Such actions will help reduce the negative health impacts of low physical activity on women and support overall societal health and gender equality.

This study also found that, although women currently bear a larger burden, the burden of low physical activity among men in China is increasing more rapidly. The number of deaths, Death rates, DALY numbers, and DALY rates for men are approaching those of women. This is mainly due to significant changes in men’s work and lifestyles, with more men working in offices, leading to increased sedentary behavior. According to the “China Male Health Survey Report,” in 2019, 60% of Chinese men sat for more than 8 h at work, an increase of about 20% from 2000. In comparison, about 50% of women sat for more than 8 h at work in 2019 (32, 33). Changes in travel patterns also play a role. In 2019, 70% of men walked less than 30 min, compared to about 50% of women (32, 33). These trends indicate that the impact of low physical activity on men and women is converging, posing new challenges for public health policy-making.

In terms of age differences, the study found that young people are less affected by low physical activity. Before the age of 25, DALYs and Death rates due to low physical activity are almost negligible. However, from the age of 25 onwards, the negative effects of insufficient physical activity become more pronounced, with DALYs and Death rates increasing as people age, and the number of affected individuals growing. This impact is especially noticeable by the age of 60, with a rapid increase in DALYs and Death rates. This is due to the gradual decline in muscle strength with age. Nilwik et al. found that muscle strength decreases by about 1 to 2% per year starting at age 40, increasing to 1.5 to 3% per year by age 60, alongside an annual loss of 1 to 2% of motor neurons. Additionally, people over 60 have about 30 to 40% fewer type II muscle fibers compared to younger individuals, and these fibers are crucial for maintaining muscle strength (34, 35). By age 70, the rate of muscle strength decline accelerates further, dropping by 3 to 5% per year. This continuous decline severely affects the daily activities of the older adult, limiting their ability to exercise. Furthermore, bone density decreases with age, and basal metabolic rate drops by an average of 5 to 10% per decade after age 50. Consequently, the risk of obesity increases with age, as does the likelihood of developing chronic diseases such as diabetes, arthritis, and heart disease (36, 37). Multimorbidity is also prevalent among the older adult, with about 70% of those over 70 suffering from two or more chronic diseases (38). These changes mean that the older adult need to invest more effort in managing their health, which further limits their ability and willingness to engage in physical activity. Therefore, China should focus more on the older adult, increasing policy and social support. This requires joint efforts from the scientific community and all sectors of society to help the older adult maintain their health.

The study also highlighted the need for special attention to the older adult population over 80 years old in China. The burden of low physical activity among Chinese people under 80 is below the global average. However, from the age of 80, this burden increases significantly, surpassing the global average. This change is primarily due to shifts in China’s population structure. The 2020 Chinese census data shows that the population over 80 years old reached 32 million, accounting for 2.26% of the total population, compared to only 6.7 million (0.58%) in 1990 (39). Notably, the study found a significant increase in the burden among males aged 85 to 94, even higher than that of females. This may be because men in this age group were working during the early stages of China’s reform and opening up, when large-scale infrastructure projects required high-risk and physical labor, leading to poorer health outcomes in old age (40). Additionally, although China’s public health system has improved significantly over the past decades, there are still considerable imbalances in resource allocation, especially in remote and rural areas. Older adult men in these regions have not received timely and effective health management (41). Therefore, the government needs to focus more on the older adult, particularly by enhancing the allocation of public sports and health resources in remote and rural areas. Encouraging these groups to participate more in physical activities and developing targeted exercise prescriptions can help mitigate the negative impacts of low physical activity.

This study has several limitations. First, while this study adopted the GBD framework’s definition of low physical activity, which provides a comprehensive assessment framework, the use of different cut-off points (such as WHO’s standard of 150 min of moderate-intensity activity per week) might yield varying estimates of disease burden. Particularly for specific populations such as the older adult, even lower-intensity physical activities might confer health benefits. Future research should consider sensitivity analyses using different threshold criteria to better understand their impact on burden estimates across various demographic groups. Second, the analysis of policies in this study did not thoroughly examine the specific implementation outcomes and mechanisms of different policies. Additionally, the lack of provincial-level data in the GBD database prevented a comprehensive spatial analysis across different regions of China. Finally, although robust statistical models (EAPC and BAPC) were employed for analysis and prediction, the accuracy of these predictions was limited by the inherent uncertainty in long-term forecasting and the inability to validate against future data. While the models effectively capture historical trends, they cannot fully account for potential future policy changes, unexpected events, or shifts in population behavior. Therefore, these projections should be interpreted as plausible scenarios rather than definitive forecasts.

## 5 Conclusion

This study is the first to reveal the disease burden caused by low physical activity in China. Although the age-standardized DALYs and Death rates due to low physical activity show a slight overall downward trend, the growth of the population and increasing aging are expected to lead to a significant increase in DALYs and deaths by 2035. Particular attention needs to be paid to diseases such as Diabetes mellitus and ischemic heart disease, as their Death and DALY rates have risen significantly. From the perspectives of age and gender, the health impact of low physical activity intensifies with age, particularly for those over 80, whose burden far exceeds the global average. The study also found that the impact of low physical activity is greater on women than on men. However, in the 85–94 age group, the burden on men significantly increases, even surpassing that of women. In conclusion, low physical activity requires more attention and the implementation of effective health interventions.

## Data Availability

The original contributions presented in the study are included in the article/supplementary material, further inquiries can be directed to the corresponding author.

## Footnotes

^1^ https://github.com/zca19990615/GBD-database
